# Hospitalizations for heat-stress illness varies between rural and urban areas: an analysis of Illinois data, 1987–2014

**DOI:** 10.1186/s12940-017-0245-1

**Published:** 2017-04-07

**Authors:** Jyotsna S. Jagai, Elena Grossman, Livia Navon, Apostolis Sambanis, Samuel Dorevitch

**Affiliations:** 1 Division of Environmental and Occupational Health Sciences, School of Public Health, University of Illinois at Chicago, Chicago, USA; 2 Centers for Disease Control and Prevention, Illinois Department of Public Health, Chicago, USA

**Keywords:** Heat stress illness, Temperature, Climate change, Temperature-heat stress illness relationship, Urban-rural differences

## Abstract

**Background:**

The disease burden due to heat-stress illness (HSI), which can result in significant morbidity and mortality, is expected to increase as the climate continues to warm. In the United States (U.S.) much of what is known about HSI epidemiology is from analyses of urban heat waves. There is limited research addressing whether HSI hospitalization risk varies between urban and rural areas, nor is much known about additional diagnoses of patients hospitalized for HSI.

**Methods:**

Hospitalizations in Illinois for HSI (ICD-9-CM codes 992.x or E900) in the months of May through September from 1987 to 2014 (*n* = 8667) were examined. Age-adjusted mean monthly hospitalization rates were calculated for each county using U.S. Census population data. Counties were categorized into five urban-rural strata using Rural Urban Continuum Codes (RUCC) (RUCC1, most urbanized to RUCC5, thinly populated). Average maximum monthly temperature (°C) was calculated for each county using daily data. Multi-level linear regression models were used, with county as the fixed effect and temperature as random effect, to model monthly hospitalization rates, adjusting for the percent of county population below the poverty line, percent of population that is Non-Hispanic Black, and percent of the population that is Hispanic. All analyses were stratified by county RUCC. Additional diagnoses of patients hospitalized for HSI and charges for hospitalization were summarized.

**Results:**

Highest rates of HSI hospitalizations were seen in the most rural, thinly populated stratum (mean annual summer hospitalization rate of 1.16 hospitalizations per 100,000 population in the thinly populated strata vs. 0.45 per 100,000 in the metropolitan urban strata). A one-degree Celsius increase in maximum monthly average temperature was associated with a 0.34 increase in HSI hospitalization rate per 100,000 population in the thinly populated counties compared with 0.02 per 100,000 in highly urbanized counties. The most common additional diagnoses of patients hospitalized with HSI were dehydration, electrolyte abnormalities, and acute renal disorders. Total and mean hospital charges for HSI cases were $167.7 million and $20,500 (in 2014 US dollars).

**Conclusion:**

Elevated temperatures appear to have different impacts on HSI hospitalization rates as function of urbanization. The most rural and the most urbanized counties of Illinois had the largest increases in monthly hospitalization rates for HSI per unit increase in the average monthly maximum temperature. This suggests that vulnerability of communities to heat is complex and strategies to reduce HSI may need to be tailored to the degree of urbanization of a county.

## Background

Illness associated with heat is the most common cause of weather-related deaths in the United States [1]. Heat-stress illness (HSI) ranges in severity from relatively mild heat cramps to life-threatening heat stroke [2, 3]. HSI was the primary cause of environmental exposure-related injuries treated in emergency departments in the U.S. from 2001 to 2004 [4]. Estimates of total health care costs based on Medicare claims data associated with hyperthermia were more than $36 million for 2004–2005 [5]. The observed rise in temperature over the past fifty years in the U.S. [6] and globally [7] is expected to continue this century. Consequently, the HSI disease burden is expected to increase.

High ambient temperature is associated with mortality [8], though most of the studies that describe that association focus on urban areas [9, 10] or urban heat waves [11–18]. Studies have typically described a non-linear relationship between temperature and mortality rate however, there is much variation based on location [10, 19]. This spatial heterogeneity suggests that populations may adapt to regional norms. Areas unaccustomed to heat, such as cities in the north, are more vulnerable and demonstrate higher mortality rates at lower absolute temperatures [10, 20]. These relationships also vary by confounding factors such as population density, socioeconomic status, and access to air conditioning [21]. The elderly, very young, and those with underlying medical conditions are the most vulnerable to heat-related mortality [22–24].

Similar to findings with mortality, studies have shown a non-linear relationship between temperature and morbidity [17, 23, 25–27]. These studies have focused on a variety of outcomes impacted by heat, including cardiovascular disease, respiratory diseases, and asthma [25, 27–29]. Recent studies utilizing emergency dispatch data demonstrated that morbidity rates are strongly associated with temperature increases [30–32]. Among the elderly, emergency department visits and hospitalizations for a variety of health conditions are more frequent during extreme heat events [25, 33–35]. Less research has focused on morbidity due to HSI itself, as opposed to morbidity due to other conditions impacted by heat [33, 36].

While research on heat-related morbidity has focused on urban areas, agricultural workers appear to be vulnerable to heat-related mortality [37] and morbidity [38, 39] due to their increased exposure to high temperatures, and perhaps the physical exertion that is required by their jobs. The rate of heat-related occupational fatalities is approximately 20 times higher for crop production workers than for U.S. workers overall (0.39 vs. 0.02 per 100,000 workers) [37]. Nevertheless, there has been limited research focusing on the variability in HSI between urban and rural areas. A study looking at HSI in North Carolina found the highest rates of morbidity and strongest associations with increasing temperature in the most rural areas studied [40]. Similarly, a study using Environmental Public Health Tracking Data found higher rates of emergency department visits for HSI in rural areas in all 14 states considered [41]. Limited research on heat morbidity in rural areas may be due to lack of adequate data in rural areas and also due to an interest in the urban heat island effect [23].

In this study, we use health, weather, and urbanization data from the state of Illinois to assess differences in associations between summer temperatures and hospitalizations for HSI as a function of urbanization. We also characterize clinical and economic aspects of HSI hospitalizations. Characterizing the relationship between temperature and urbanization on HSI rates will improve our understanding of the ambient temperature-HSI morbidity relationship and it can be useful for public health preparedness efforts.

## Methods

### Hospitalization data

We utilized hospital discharge data from the Illinois Department of Public Health (IDPH) Hospital Discharge Database. The database includes discharge data from 97% of Illinois hospitals; the remaining non-member hospitals are located in Cook County, a primarily urban area encompassing the city of Chicago, and account for 7% of all Cook County hospitalizations [42]. Each record includes patient demographics, county of residence, and up to nine diagnoses codes. Diagnoses were coded using the International Classification of Diseases, 9th Revision, Clinical Modification (ICD-9-CM). Heat-related illness was defined as including diagnosis of ICD-9-CM 992.x, effect of heat or light, that includes heat stroke, heat syncope, and heat exhaustion, or ICD-9-CM E900, accidents due to heat as any one of the diagnoses (up to nine per case) recorded. Due to the low number of cases with relevant diagnoses, data were provided aggregated monthly. Cases were limited to the summer months, May through September, for the 28-year period from 1987 to 2014.

**Fig. 1:**
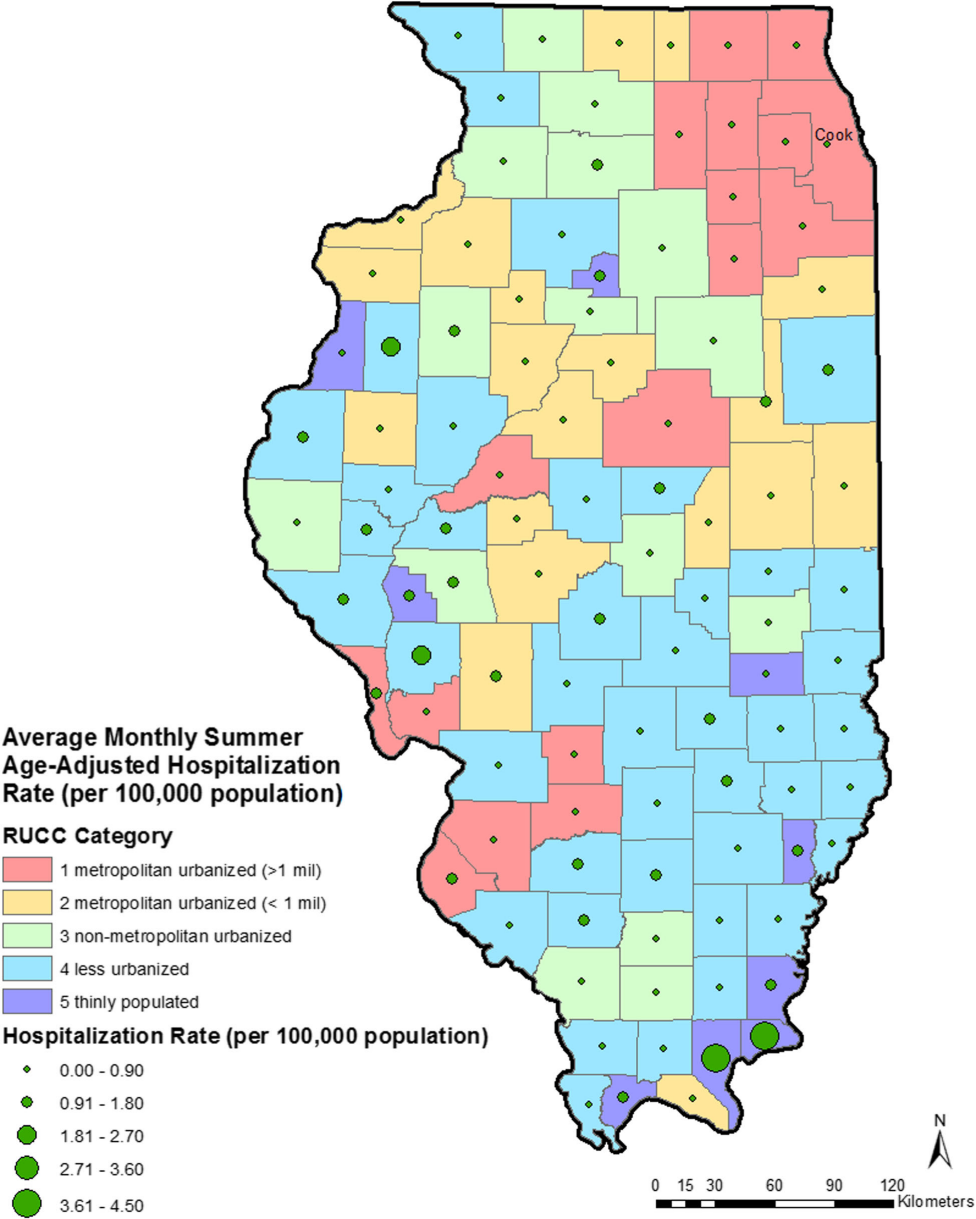
Map of Illinois showing urban/rural classifications and average monthly summer age-adjusted hospitalization rate for heat-stress illness (per 100,000 population) by county for the 28-year study period. Cook: Cook County, that contains the City of Chicago

Age-adjusted mean summer monthly HSI hospitalization rates were calculated per 100,000 population for each county by year using direct standardization methods. To account for changes in the population over the 28-year study period, the study period was divided into three time periods and corresponding population data were used to calculate rates. For the years 1987–1994, the 1990 Census data [43] was used as the denominator, for years 1995–2004, the 2000 Census data [43] was as the denominator, and for the years 2005–2014, the 2010 Census data [43] was used as the denominator. Nine age categories were created, 0–9 years, 10–19 years, 20–29 years, 30–39 years, 40–49 years, 50–59 years, 60–69 years, 70–79 years, and ≥80 years of age. Average summer monthly HSI hospitalization rates by county are shown in Fig. 1.

### Urban/rural stratification

Counties were categorized into five urban/rural strata using Rural Urban Continuum Codes (RUCC) [44]. The original nine continuum codes were aggregated into five strata for this analysis: RUCC1 (original RUCC1), metropolitan urbanized, (>1 million population); RUCC2 (original RUCC2 and RUCC3), metropolitan urbanized (< 1 million population); RUCC3 (original RUCC4 and RUCC5), non-metropolitan urbanized; RUCC 4 (original RUCC6 and RUCC7), less urbanized; RUCC5 (original RUCC8 and RUCC9), thinly populated.

### Temperature data

**Fig. 2:**
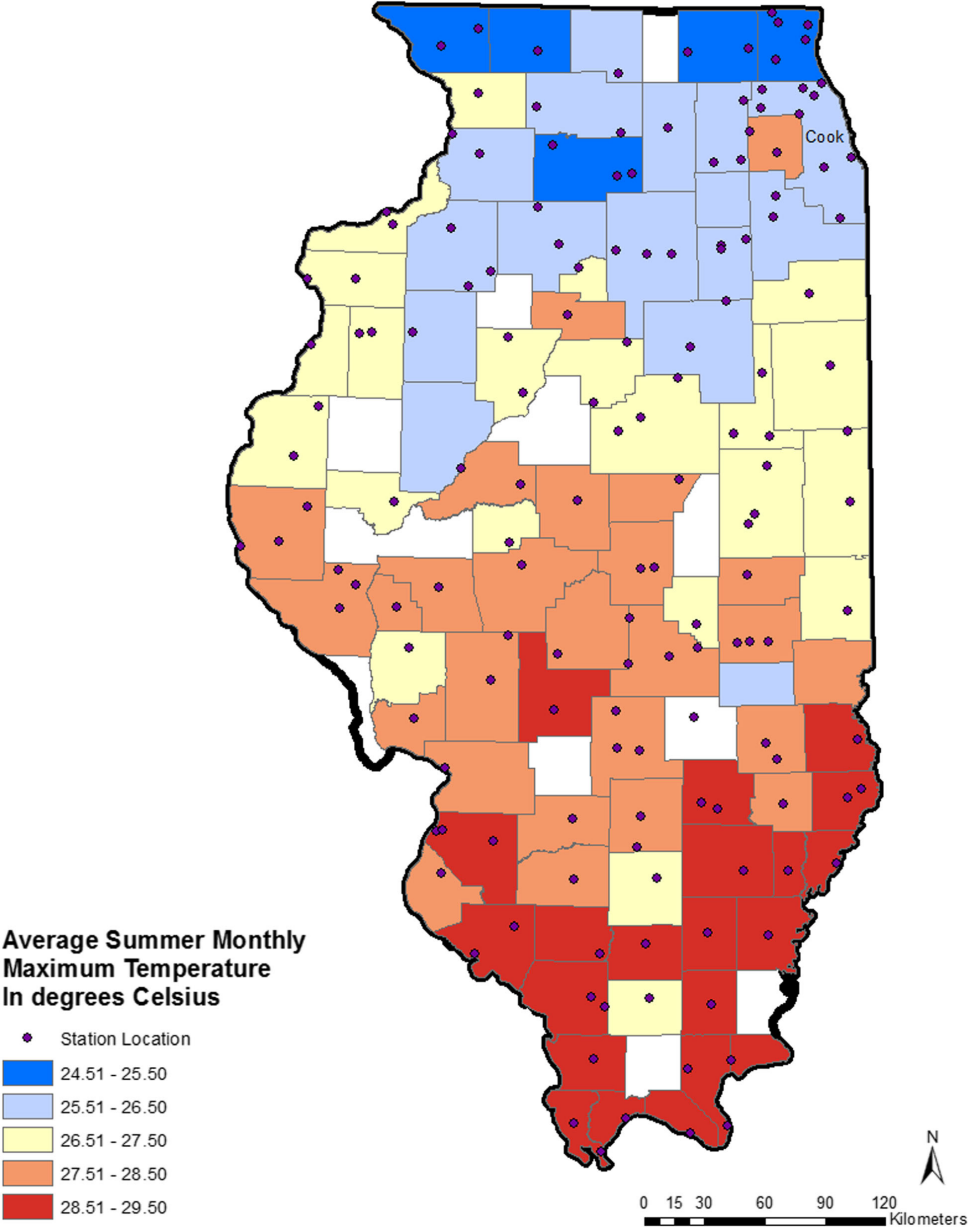
Map of Illinois showing annual average summer monthly maximum temperature in degrees Celsius by county and locations of weather stations. Cook: Cook County, that contains the City of Chicago

Daily weather data for the 28-year study period were obtained from the Midwest Regional Climate Center [45]. These data were collected from 235 weather stations located throughout the state of Illinois. Of these, 81 stations only provided data on precipitation and were not used for this analysis. The remaining 154 stations provided data on temperature for some or all of the 28-year study period. Most counties had at least one station that provided data and several had more than one station (Fig. 2). Counties without a monitoring station were assigned temperature from the nearest monitoring station. Data collected at each station included daily maximum, minimum, and mean temperature. Utilizing all available data for the county, we calculated average monthly maximum temperature, minimum temperature, and mean temperature, in degrees Celsius, for each summer month, May–September, in the study period.

### Covariate data

For each Illinois county, we complied the percent of population below the poverty level, percent of population that was non-Hispanic Black, and percent of population that was Hispanic [43]. Covariate data for the years 1987–1994, 1995–2004, and 2005–2014 were obtained from the 1990, 2000, and 2010 Census, respectively.

### Statistical analysis

We assessed associations between monthly average maximum temperature and age-adjusted monthly rates of hospitalizations for HSI at the county-level. All analyses were stratified by the five urbanization strata. The association between monthly mean temperature with monthly county hospitalization rates for HSI were assessed using random slope, random intercept multi-level linear regression models. County of residence was considered a fixed effect and temperature as the random effect (Eq. 1). In addition, analyses were adjusted for county level covariates including, percent of population below the poverty level, percent of the county population that was non-Hispanic Black, and percent of population that was Hispanic.

1 $$ {Y}_{i j}={\beta}_0+{\beta}_1{X}_i+{b}_{0 i}+{b}_{1 i}{x}_{i j} $$

Where:

*Y*_*ij*_ = outcome rates (HSI hospitalization) for the *i* ^*th*^ county and the *j* ^*th*^ month,

*X*_*i*_ = indicator variable for the *i* ^*th*^ county (fixed effect),

*x*_*ij*_ = exposure measurement (mean temperature) for the *i* ^*th*^ county and the *j* ^*th*^ month (random effect).

In this model *X*_*i*_ represents a fixed effect for each county and *x*_*ij*_, the mean temperature, accounts for a random effect. Models were also adjusting for county-level population below the poverty level, percent of the county population that was non-Hispanic Black, and percent of population that was Hispanic.

Results are reported as a change in hospitalization rate per 1 °C change in maximum monthly average temperature and 95% confidence intervals (CIs). To evaluate whether urban/rural differences in HSI rates might be attributable to a severe heat wave in 1995 that impacted much of the Midwest, including Illinois, and resulted in an estimated 739 fatalities [18] in the most populous urban county in Illinois (Cook County), analyses were conducted with and without 1995 data. Analyses were conducted using R version 3.3.1 (R Foundation for Statistical Computing) and SAS (SAS Institute, Cary, NC), version 9.4.

This work, that involved the analysis of a dataset that had personal identifiers removed or grouped into categories (such as age) was determined not to involve human research subjects by the UIC Institutional Review Board (Protocol 2013-0172).

### Clinical and economic analyses

Comorbidities of individuals hospitalized for HSI were identified using the nine additional ICD-9-CM diagnosis codes. Conditions that are clearly chronic (essential hypertension, diabetes) were not considered. Those that are acute (syncope or fainting; dehydration with electrolyte abnormality), chronic but potentially worsened by heat stress, or acute due to heat stress (renal insufficiency, atrial fibrillation) were considered. Furthermore, some conditions (urinary tract infection) may have developed after the patient had been admitted to the hospital. The hospital discharge dataset contained information about length of stay and charges for each admission that we tabulated. We used the U.S. Bureau of Labor Statistics data to convert hospital charges to 2014 dollars [46].

## Results

From 1987 to 2014, 8856 hospitalizations for HSI occurred in Illinois in the months of May through September. Of those, 136 were not residents of Illinois (primarily these individuals were residents of Missouri or Indiana) and 148 were residents of Illinois with an unknown county of residence, leaving 8667 residents of Illinois whose county of residence was known. All analyses described herein are limited to these 8667 cases. The annual mean number of HSI hospitalizations was 308 cases (range: 69–1511). Excluding 1995, the annual mean was 254 cases (range: 69–508). Males accounted for 5322 (61.7%) of cases. The largest number of HSI hospitalizations and highest rate was seen in those aged ≥80 years (Table 1). Within each age category, rates of hospitalizations generally increased within decreasing urbanicity. However, in the older age category, 80+ years, the highest rates were seen in the metropolitan (>1 million population) counties.

**Table 1:** Number and overall rate of summer hospitalizations of heat-stress illness (per 100,000 population) over the 28-year study period by rural/urban stratification (RUCC1–metropolitan urbanized (> 1 million population); RUCC2–metropolitan urbanized (< 1 million population); RUCC3–non-metropolitan urbanized; RUCC 4–less urbanized; RUCC5–thinly populated) and by age categories

RUCC	Counties (N)	<10y Count (rate)	10–19y Count (rate)	20–29y Count (rate)	30–39y Count (rate)	40–49y Count (rate)	50–59y Count (rate)	60–69y Count (rate)	70–79y Count (rate)	≥80 y Count (rate)	All Count (rate)
RUCC1 – metropolitan, (> 1 million pop.)	17	79 (0.05)	270 (0.16)	137 (0.07)	409 (0.22)	614 (0.36)	706 (0.53)	726 (0.79)	1114 (1.87)	1454 (4.11)	5510 (0.453)
RUCC2 – metropolitan (< 1 million pop.)	19	26 (0.09)	79 (0.25)	53 (0.17)	78 (0.25)	123 (0.39)	157 (0.60)	151 (0.78)	176 (1.30)	187 (2.18)	1029 (0.458)
RUCC3 – non-metropolitan urbanized	15	7 (0.05)	61 (0.37)	38 (0.24)	68 (0.43)	82 (0.50)	111 (0.80)	74 (0.67)	124 (1.51)	141 (2.61)	706 (0.594)
RUCC4 – less urbanized	42	16 (0.08)	121 (0.57)	58 (0.31)	155 (0.74)	155 (0.70)	178 (0.96)	181 (1.20)	194 (1.71)	268 (3.61)	1325 (0.850)
RUCC5 – thinly populated	9	0 (0.00)	8 (0.71)	11 (1.26)	11 (1.08)	12 (1.04)	11 (1.07)	15 (1.61)	17 (2.48)	11 (2.86)	98 (1.158)
All	102	128 (0.05)	539 (0.22)	298 (0.12)	721 (0.28)	986 (0.41)	1164 (0.60)	1146 (0.83)	1624 (1.74)	2061 (3.60)	8667 (0.503)

HSI hospitalization rates varied substantially across counties (Fig. 1). The mean summer age-adjusted rate per county ranged from 4.46 cases/100,000 population (Pope County) to <1 case/100,000 population (16 counties). Cook County, that contains the City of Chicago, accounted for 3731 (44.6%) of all cases over the 28-year period, but the lowest age-adjusted rate (0.13/100,000 population). Nearly half (49.1%) of HSI hospitalizations among Illinois residents occurred during the month of July; excluding data from 1995 that percent was 42.6%. In general, the highest summer temperatures are seen in the southern part of the state, which is also the least urbanized area of Illinois (Fig. 2).

**Fig. 3:**
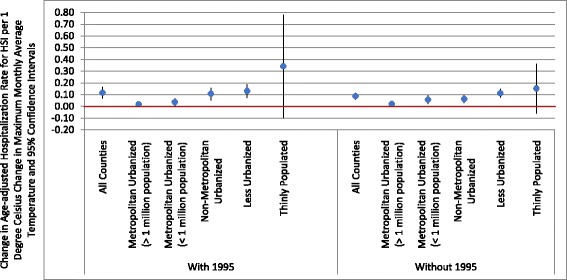
Estimated change in heat-stress illness per 1 °C change in maximum monthly average temperature (after adjustment for county percent below poverty level, percent population Black, and percent population Hispanic) and 95% confidence intervals for all counties and by rural/urban stratification (RUCC1–metropolitan urbanized (> 1 million population); RUCC2–metropolitan urbanized (< 1 million population); RUCC3–non-metropolitan urbanized; RUCC 4–less urbanized; RUCC5–thinly populated) for all study years, 1987–2014, and excluding 1995, the year of the heatwave

A significant association between mean monthly maximum temperature and HSI hospitalization rates by county was observed for Illinois overall (Fig. 3). For Illinois counties overall, a 1 °C increase in maximum monthly average temperature was associated with a 0.12 increase in rate of hospitalizations per 100,000 population (95% Confidence Interval (CI): 0.07, 0.17). The strongest association, though not significant at a *p* = 0.05 level, was seen in the most thinly populated stratum, where a 1^○^C increase in maximum monthly average temperature was associated with a 0.34 (95% CI: −0.10, 0.78) increase in HSI hospitalization rate per 100,000 population. By comparison, a 1^○^C increase in maximum monthly average temperature was associated with a 0.02 (95% CI: −0.003, 0.04) increase in HSI hospitalization rate per 100,000 population in the metropolitan urban (> 1 million population) stratum. Excluding the 1995 data had minimal impact on the monthly temperature-monthly hospitalization association, with the exception of the thinly populated strata, that demonstrated a large decrease in the effect estimate (Fig. 3).

Sensitivity analysis included an evaluation of associations with monthly minimum temperature and mean monthly temperature. Due to the high correlation between maximum, minimum and mean temperature, there was minimal change in effect estimates and the overall trends demonstrated remained the same (data not shown).

The mean and median length of an HSI hospital stay was 3.7 and 2.0 days, respectively (range: 1–122 days). Total and mean per person hospital charges in 2014 dollars were $167.7 million and $20,050, respectively. The most common concurrent diagnoses among HSI cases were dehydration, electrolytes/acid-base abnormalities, and acute renal failure (Table 2). Other concurrent conditions known to be associated with heat stress include syncope and rhabdomyolysis. Rhabdomyolysis, that can lead to multisystem organ failure, was more common in the younger age categories (chi square *p* < 0.0001). While individuals <50 years of age accounted for 26.2% of HSI hospitalizations overall, they accounted for 50.3% of HSI cases complicated by rhabdomyolysis. In addition, several chronic conditions were common among cases of HSI, hypertension (27%), diabetes (8.8%), congestive heart failure (7.4%), and chronic obstructive pulmonary disease (5.1%).

**Table 2:** Most common co-morbid diagnoses listed with hospitalizations for heat-stress illness in Illinois, 1984–2014. Note that percentages do not sum to 100% as hospitalized patients typically had multiple diagnoses

Diagnosis	ICD-9 codes	Frequency (percent)
Dehydration	276.5, 276.51, 276.52	3694 (42.6)
Electrolyte, acid-base abnormality	276.1, 276.2, 276.3, 276.4, 276.7, 276.8, 276.9	1445 (16.7)
Acute renal failure	584, 584.5, 584.6, 584.7, 584.8, 594.9	1021 (11.8)
Urinary tract infection	599.0	808 (9.3)
Syncope (fainting)	780.2	781 (9.0)
Rhabdomyolysis	728.88	549 (6.3)
Atrial fibrillation	427.31	514 (5.9)
Respiratory failure	518.81	299 (3.5)
Altered mental status	293.0, 780.02	92 (1.1)

## Discussion

We assessed the association between the average maximum monthly temperature and rates of HSI hospitalization among Illinois counties. We utilized data for at 28-year study period, 1987–2014 focusing on the summer seasons (May to September). Previous epidemiological literature has focused on urban areas. Our study covers the entire state of Illinois and therefore we were able to assess differences by urban/rural strata.

We collapsed the nine rural urban continuum codes [44] into five strata to assess the relationship between mean monthly maximum temperature and hospitalizations for HSI along the continuum of urban metropolitan to thinly populated areas. In Illinois, we found that HSI hospitalization rates and the temperature-hospitalization response function was the strongest in the thinly populated strata. This stratum is the most isolated strata from the urban and suburban centers in the state. These results are consistent with recent studies that demonstrated elevated risk in rural areas for emergency room visits for HSI across the U.S. [41, 47] and in North Carolina [40], though those studies did address hospitalization.

There are several possible factors contributing to susceptibility of the most rural areas to heat-stress illness. Studies of urban heat waves have demonstrated that living alone, lack of air conditioning, and underlying medical conditions are risk factors for mortality [13, 15]; these same factors may affect residents in rural areas. Particularly in Illinois, following the 1995 heat wave, several prevention measures were put in place in the City of Chicago, including heat warning systems, community outreach initiatives, and cooling centers [13]. Similar efforts may not have been implemented to a comparable extent in rural areas.

In Illinois, the counties identified as thinly populated are also agricultural counties. The relatively high proportion of the population engaged in physically-demanding, outdoor work in those counties may account for the steeper slope of the temperature-HSI association (Fig. 3). Our data demonstrated a more pronounced difference in rates by urban/rural strata in those less than 60 years of age, suggesting that occupational factors might place workers at risk for HSI that is consistent with previous literature [37, 38]. Previous literature regarding the use of hospitalization data has suggested that hospitalizations in rural areas may be underreported compared with urban areas [40]; therefore, the effect demonstrated in rural areas may be an underestimate of the true effect of heat in rural areas of Illinois.

Exposure to extreme heat is known to have a complex set of physiological effects on multiple organ systems. We found fluid and electrolyte balance and acute failure to be the two most common comorbidities associated with HSI in Illinois that is consistent with previous studies [48, 49]. Increased risk of hospitalization among the elderly for fluid and electrolyte disorders, renal failure, urinary tract infection, septicemia, and heat stroke heat wave days compared with non-heat wave days has been documented [50]. The costs associated with heat events can also have significant impacts. Previous analyses of the California heat wave, which lasted about 15 days, estimated the total costs to be $5.4 billion dollars (in 2008 dollars) of which $2.8 billion was the cost of hospitalizations [25, 51]. While our results are not directly comparable, since we are not evaluating a specific heat wave, we found that total hospitalizations in Illinois for HSI cost $167.7 million dollars (2014 dollars) for the 28-year study period. Additionally, the $167.7 million in hospital costs is only a portion of the total economic burden. The total of 32,564 days spent in hospitals by those with HSI represents a substantial burden of indirect costs. Improved and targeted awareness campaigns regarding the health impacts of heat may reduce the burden of associated healthcare costs.

Our analysis is limited in a few ways. First, the number of HSI hospitalizations is likely to be underestimated. Prior studies have identified HSI hospitalizations as we have, based on either an ICD-9-CM code of 992 or E900 or both [12, 52]. However, a limitation of this approach is that health care providers must recognize that an individual’s symptoms – that can be non-specific – are due to heat in order for the condition to be coded as being heat-related. Morbidity due to a variety of health conditions, including renal, cardiac, and respiratory diagnoses can increase with heat. A study of Medicare beneficiaries found that only a small subset of excess heat-related hospitalizations were coded with ICD-9 codes 992 or E900 [53]. Thus, although these ICD-9 codes may be relatively specific for HSI they may have limited sensitivity for the broad range of illness exacerbated or caused by heat exposure when the role of ambient heat is not recognized by clinicians. Therefore, the estimated 8667 cases of HSI hospitalizations described here are likely an underestimate of the burden of disease attributable to ambient heat in Illinois during 1987–2014. Due to the low number of hospitalizations, we were provided monthly aggregated data that limited the types of analyses possible. We were could not consider a case-crossover analysis which would have been appropriate for this acute and rare outcome. In addition, we did not have the statistical power required to consider daily associations but needed to aggregate by month. Therefore, we were not able to assess lagged associations between temperature and hospitalizations for HSI but rather, were only able to assess monthly associations. Second, maximum temperature exposure was based on county of residence identified in the hospitalization dataset. It is possible that heat exposure may have occurred in a different county. However, this would bias our results to the null and we expect this error to be minimal in Illinois as neighboring counties do not experience significantly different temperature patterns. A third limitation is the use of hospital charges to estimate the direct medical costs of HSI hospitalizations. Billed hospital charges are inflated above cost, and thus, do not accurately capture the direct medical costs of HSI hospitalizations in Illinois. Fourth, it is not known to what degree general disparities in access to hospital care in rural vs. urban areas may contribute to the differences in HSI hospitalizations among Illinois counties.

Reduction of HSI risk in thinly population rural areas may require different strategies than in urban areas, given the low population density and high prevalence of agricultural work. Input from emergency preparedness specialists would be useful in identifying effective methods for alerting people, particularly those working in agriculture, with timely updates from local offices of the National Weather Service. Such alerts might include reminders for rest, water, and shade breaks. County health departments in rural areas, organizations that address health and social needs in rural areas, and agricultural extension offices could identify cooling centers and promote their use if such initiatives would be shown to be acceptable to local communities. Educational initiatives for rural health care providers could include methods to increase awareness among residents, to increase fluid intake during hot weather. Given the high frequency of acute renal failure and rhabdomyolysis, promoting fluid intake is critical, particularly for those who engage in exertional activities. Prevention efforts should account for U.S. National Climate Assessment [54] projections of more frequent and extreme heat events.

## Conclusion

Our findings suggest that elevated temperatures may have a differential impact in rural counties compared with urban counties. Future research should be conducted at the individual level to understand specific pathways of exposure, such as occupational, that result in higher rates of HSI in rural communities. Prevention of heat-related illness in rural areas will require different strategies than urban areas, given lower population densities and higher prevalence of agricultural work. Collaboration among emergency management, community organizations, and local health departments is needed to reduce HSI risk in rural counties.

## Abbreviations

HSI: Heat-stress illness
ICD-9-CM: International Classification of Diseases, 9th Revision, Clinical Modification
RUCC: Rural Urban Continuum Codes
